# Supplementary material for: Identification of photoperiod-induced specific miRNAs in the adrenal glands of Sunite sheep (Ovis aries)
Source: Front Vet Sci. 2022 Jul 22;9:888207. doi: 10.3389/fvets.2022.888207 (PMC9354845; doi:10.3389/fvets.2022.888207)
Supplement: Supplementary file 1 [file Data_Sheet_1.ZIP › Supplementary file/miRDeep2 file.zip!/miRDeep2 file/Know_Structure/LP42A3/oar-mir-376d.pdf]

miRBase precursor : oar-mir-376d
Total read count : 159
oar-miR-376d read count : 155
remaining reads : 4

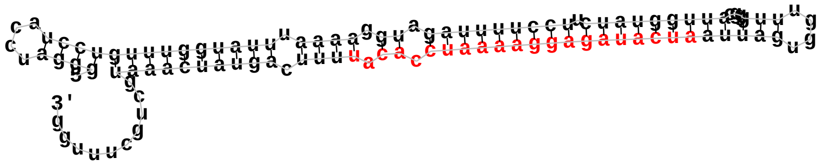

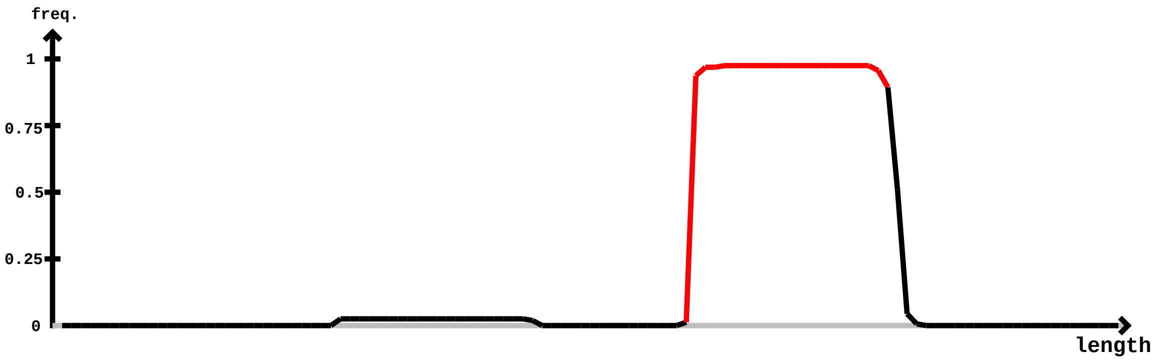

oar-miR-376d

```
5'- gggauccauccuguuugguauuuaaaagguagauuuuccuucuaugguuacggguuuggugauuaaucauagaggaaaauccacauuuucaguaucaaaugcugcuuugg   -3'    exp
    ((((....))))((((((((((.((((.((.(((((((((.((((((((...(((....))))))))))))))))))))))).)).)))).))))))))))............   reads      mm         sample
    ............................guagauuuuccuucuauggu..............................................................   1          0          seq
    ............................guagauuuuccuucuaugguu.............................................................   3          0          seq
    ................................................................aaucauagaggaaaauccaca.........................   2          0          seq
    .................................................................aucauagaggaaaauccac..........................   3          0          seq
    .................................................................aucauagaggaaaauccaca.........................   8          0          seq
    .................................................................aucauagaggaaaauccacau........................   61         0          seq
    .................................................................aucauagaggaaaauccacauu.......................   71         0          seq
    .................................................................aucauagaggaaaauccacauuu......................   3          0          seq
    .................................................................aucauagaggaaaauccacauuuu.....................   1          0          seq
    ..................................................................ucauagaggaaaauccacauu.......................   2          0          seq
    ..................................................................ucauagaggaaaauccacauuu......................   3          0          seq
    ....................................................................auagaggaaaauccacauu.......................   1          0          seq
```
